# Programmed death-ligand 1 expression in surgically resected thymomas

**DOI:** 10.1007/s13304-025-02242-w

**Published:** 2025-05-25

**Authors:** Luca Frasca, Antonio Sarubbi, Filippo Longo, Valentina Marziali, Alexandro Patirelis, Pierfilippo Crucitti, Vincenzo Ambrogi

**Affiliations:** 1 Department of Thoracic Surgery, Fondazione Policlinico Universitario Campus Bio-Medico, Via Alvaro Del Portillo 200, 00128 Rome, Italy; 2 Doctoral School of Microbiology, Immunology, Infectious Diseases and Transplants (MIMIT), Tor Vergata University, Viale Oxford 81, 00133 Rome, Italy; 3 Department of Thoracic Surgery, Tor Vergata University Polyclinic, Viale Oxford, 81, 00133 Rome, Italy

**Keywords:** Thymic epithelial tumours, Thymoma, PD-L1, Thymectomy, Recurrence

## Abstract

Thymomas are one of the most common neoplasms of the anterior mediastinum with limited therapeutic options, particularly in advanced stages. The molecular profiles of these tumors remain poorly investigated. This study aims to evaluate the expression of programmed death ligand 1 (PD-L1) in a selected cohort of intentionally curative resected thymomas and evaluate a possible relationship with the risk of recurrence. This retrospective bicentric study analyzed a group of patients who underwent complete thymectomy with curative intent. PD-L1 expression was assessed through immunohistochemistry using the Ventana PD-L1 assay. PD-L1 expression was assessed as low if <50% or high when ≥50%. The Kaplan–Meier method and Cox regression analysis were performed to evaluate a possible relationship between PD-L1 expression and disease-free survival. High PD-L1 expression was observed in 46.2% of patients. Overexpression of this protein was significantly associated with aggressive (B2/B3) thymomas histotypes (p<0.001). During follow-up period, 12/52 patients developed recurrence. High PD-L1 expression correlated with reduced disease-free survival with Kaplan-Meier method (p<0.001). Furthermore, PD-L1 expression more than 50% resulted to be related to a worse prognosis at multivariable Cox regression analysis (hazard ratio 5.4, 95% confidence interval 1.5–16.9, p=0.028) together with histology (p=0.044) and Masaoka-Koga stage (p=0.026). The elevated expression of PD-L1, particularly in aggressive thymoma subtypes, underscores its potential as a prognostic biomarker. These findings support the need for further research to explore the potential of immunotherapy in treating these rare malignancies.

## Introduction

Thymic epithelial tumours represent the most common neoplasms of the anterior mediastinum and thymoma is the most predominant histotype within this group [1]. The incidence ranges from 1.3 to 3.2 per million, with a mean age at diagnosis from 50 to 60 years [1]. Thymomas can present with a wide spectrum of symptoms. In fact, 30–40% of patients with thymoma have coexisting autoimmune disorders, particularly Myasthenia Gravis [2].

In early stages radical surgery is the gold standard treatment and it is the most important clinical factor influencing both recurrence rates and prognosis. Unfortunately, 20–30% of thymomas are unresectable or metastatic at the time of diagnosis [3]. The standard therapy for these patients is chemotherapy alone or with radiotherapy although with limited results and significant side-effects [3].

For this reason, after positive results achieved in other neoplasms, antibodies against Programmed Death Ligand 1 (PD-L1) could represent a valid integration or an alternative option for more aggressive thymomas [4, 5]. However, because of the biological characteristics of the thymus, immunotherapy can cause serous immune-related adverse events [6, 7].

Despite the growing interest in this alternative therapeutic approach, many studies previously conducted on immunotherapy in thymomas reported discordant conclusions [8–10]. In particular, there is actually no consensus about the necessity of adjuvant treatments in patients with thymoma after surgery without residual disease even if some of these tumors present a more aggressive behaviour.

The aim of this study is to analyse PD-L1 expression in surgically resected thymomas [11], trying to find whether its 50% expression, similarly to other neoplasms, could be considered a marker of recurrence.

## Materials and methods

### Study population

We retrospectively reviewed medical records of patients undergoing surgical resection for thymoma in two major centres devoted to mediastinal surgery between September 2014 and January 2019. Multidisciplinary evaluations and follow-up periods ranging from 1 to 5 years enabled detailed analysis of treatment outcomes and disease progression. The study was conducted with the approval of the Internal Review Board (approval n 262.24).

### Study design

Patients eligible for the study were those who underwent complete thymectomy (removal of the tumour and entire thymus) with a final histological report consistent with thymomas [12]. Exclusion criteria comprised patients with thymic carcinoma, clinical stage IV thymoma, thymic hyperplasia, Castleman disease, and thymic plasmacytoma. Additional filtering was applied to exclude patients who were followed by other centers after surgery, not providing information about their status after at least 12 months. Conversely, patients who were lost during the first year of follow-up due to death or recurrence were included in the study.

### Preoperative evaluation

Pre-operative staging was performed by thoracic computed tomography (CT) scan and 18-fluorodeoxyglucose-positron emission tomography (18-FDG-PET) scanning. Images were obtained within a maximum timeframe of 30 days before the surgical intervention [3]. Furthermore, the diameter of the neoplastic lesions was precisely calculated at CT scans, with a cut-off point of 40 mm established for classification purposes [13]. PET value was divided according to standard uptake value (SUV)max cut-off point of 3 [14].

### Surgical procedure

All patients underwent complete thymectomy (en bloc resection of the tumour, entire thymus tissue, and upper poles). In cases of myasthenia gravis, an extended thymectomy also including the mediastinal fatty tissue was accomplished to improve neurological control [15]. Surgical techniques included Uniportal Video-Assisted Thoracic Surgery (uVATS), median sternotomy, or thoracotomy, chosen based on preoperative radiological findings and patient factors [16]. Thoracotomy was always left-sided, while VATS could be performed from either side according to either the prevalent location of the tumour or the preference of the surgical team. Multidisciplinary meetings guided treatment, and adjuvant therapy was provided for advanced tumours. All patients received general anesthesia and provided written informed consent. Staging was assessed according to the Masaoka-Koga system [17]. Histology was evaluated using hematoxylin and eosin staining after formalin-fixation and paraffin-embedding and was based on World Health Organization classification [18]. PD-L1 analysis was retrospectively performed using PD-L1 immunohistochemistry on embedded samples by the VENTANA PD-L1 (SP263) assay, with positivity defined as PD-L1 expression ≥50%. This threshold was chosen according to previous literature about thymoma and also considering that it has been demonstrated to be reliable to a better response to pembrolizumab treatment in non-small-cell lung cancer in clinical practice [18]. A positive control tissue based on placenta was applied to validate the PD-L1 immunohistochemistry staining reaction.

### Statistical analysis

Statistical analysis was performed using SPSS Statistics version 26.0 (IBM Corp. Released 2016. IBM SPSS Statistics, Armonk, NY, USA: IBM Corp.). Descriptive statistics included median and interquartile range (IQR) for continuous variables, and frequencies for discrete variables.

Due to the small sample size, Fisher’s exact test was applied to compare PD-L1 as a categorical variable (<50% vs ≥50%) to the other ones. A significance level of p≤0.050 was used. Cox univariable proportional hazards regression was performed to evaluate the impact of different variables on prognosis. Those resulted significant were subsequently processed into a multivariable assay. In order to simplify the analysis, multifactorial variables were dichotomised as follows: age according to median value, surgical approach in open-access vs mini-invasive, body mass index (BMI) according to normal weight definition (≤25% vs >25%) [19], comorbidity into no versus at least one, stage into initial (stage I) versus advanced (Stage II and III), histology into low- (A, AB, B1) vs high-grade (B2,B3) [3]. Kaplan-Meier analysis evaluated the association between PD-L1 expression and disease-free survival (DFS) and was applied for subgroup analysis among patients with PD-L1 ≥ 50%. Overall survival (OS) was not analysed, as no patients died during the study period.

## Results

### Clinical and Pathological Characteristics

A total of 63 subjects consecutively underwent complete thymectomy in both centres. Seven cases resulted thymic hyperplasia, 2 thymic carcinoma, 1 Castleman’s disease, and 1 thymic plasmacytoma. Thus, the final population consisted of a cohort of 52 patients meeting all inclusion criteria. The median age of the study population was 68 years (IQR 61–74). Demographic and clinical data were reported in Table 1.

**Table 1:** Demographic and pathological characteristics of the study population

Variable	
Age (years), median (IQR)	68.0 (61.0–74.0)
Gender, n (%)	
Male	23 (44.2%)
Female	29 (55.8%)
BMI (kg/m^2^), median (IQR)	27.0 (23.1–31.4)
BMI group, n (%)	
Normal (18.5–24.99 kg/m^2^)	23 (45.1%)
Overweight (25–29.99 kg/m^2^)	15 (29.4%)
Obese (30–39.99 kg/m^2^)	13 (25.5%)
Comorbidity, n (%)	
Hypertension	21 (40.4%)
Diabetes	10 (19.2%)
Dyslipidemia	10 (19.2%)
Autoimmune disease	20 (38.5%)
Smoke history, n (%)	
No	14 (26.9%)
Yes	38 (73.1%)
CT diameter (mm), median (IQR)	45.0 (33.0–55.0)
CT diameter cut-off, n (%)	
≤40 mm	22 (42.3%)
>40 mm	30 (57.7%)
SUVmax, n (%)	
≤3	32 (61.5%)
>3	20 (38.5%)
Treatment, n (%)	
Surgery alone	35 (67.3%)
Adjuvant therapy	17 (32.6%)
Surgical approach, n (%)	
VATS	19 (36.5%)
Sternotomy	11 (21.2%)
Thoracotomy	22 (42.3%)
WHO Histology, n (%)	
A	8 (15.4%)
AB	15 (28.9%)
B1	10 (19.2%)
B2	14 (26.9%)
B3	5 (9.6%)
Masaoka-Koga Stage, n(%)	
Stage I	14 (26.9%)
Stage II	30 (57.7%)
Stage III	8 (15.4%)

*BMI* body mass index, *CT* computed tomography, *IQR* interquartile range, *SUV* standard uptake value, *VATS* video-assisted thoracic surgery, *WHO* World health organization

A history of at least one autoimmune disease was found in 20 patients (38.5%) including rheumatic fibromyalgia (n=3), undifferentiated connective tissue disease (n=1), pemphigus vulgaris (n=1), thyroiditis (n=5), cutaneous psoriasis (n=4), rheumatoid arthritis (n=6), and MG (n=3). Additionally, 70% of the patients were either current or former habitual smokers.

Thirty-five patients (67.3%) received surgery as sole treatment. Surgical approaches were u-VATS in 19 patients (36.5%), 10 from the right and 9 from the left side, respectively. Sternotomy was chosen for 11 patients (21.2%) and left-sided thoracotomy for 22 patients (42.3%). Seventeen patients (32.6%) received adjuvant therapy following surgery. Of these, 16 patients (30.8%) underwent radiotherapy and 5 patients (9.6%) received a combination of both radiotherapy and chemotherapy. The median CT tumour diameter was 45.0 mm (IQR 33.0–55.0), exceeding 40 mm in 30 patients (57.7%). Stage I tumours were identified in 14 patients (26.9%), stage II in 30 patients (57.7%), and stage III in 8 patients (15.4%). Histology was as follows : type A (n=8), AB (n=15), B1 (n=10), B2 (n=14) and B3 (n=5).

### PD-L1 results

Patients with PD-L1 expression <50% comprised 53.8% (28/52) of the entire population, while 46.2% (24/52) had PD-L1 expression ≥50%. Distribution according to clinical variables is shown in Table 2.

**Table 2:** Fisher Test results

	PD-L1 <50% (n=28)	PD-L1 ≥50% (n=24)	p-value
Gender			
Males	11 (39.3%)	12 (50.0%)	0.57
Females	17 (60.7%)	12 (50.0%)	
Age			
≤68 years	16 (57.1%)	11 (45.8%)	0.58
>68 years	12 (42.9%)	13 (54.2%)	
Smoke history			
Yes	5 (17.9%)	9 (37.5%)	0.13
** No**	23 (82.1%)	15 (62.5%)	
Comorbidity			
Yes	10 (35.7%)	15 (62.5%)	0.094
No	18 (64.3%)	9 (37.5%)	
Autoimmune disease			
Yes	11 (39.3%)	10 (41.7%)	0.89
No	17 (60.7%)	14 (58.3%)	
Myasthenia			
Yes	0 (0.0%)	3 (12.5%)	0.092
No	28 (100.0%)	21 (87.5%)	
CT Diameter			
≤40 mm	14 (50.0%)	8 (33.3%)	0.27
>40 mm	14 (50.0%)	16 (66.7%)	
SUVmax			
≤3	21 (75.0%)	11 (45.8%)	0.046
>3	7 (25.0%)	13 (54.2%)	
Treatment			
Surgery alone	27 (96.4%)	8 (33.3%)	<0.001
Adjuvant therapy	1 (3.6%)	16 (66.7%)	
Surgical approach			
VATS	13 (46.4%)	6 (25.0%)	0.15
Open	15 (53.6%)	18 (75.0%)	
BMI			
≤25 kg/m^2^	10 (35.7%)	10 (41.7%)	0.78
>25 kg/m^2^	18 (64.3%)	14 (58.3%)	
WHO histology			
A/AB/B1	26 (92.9%)	7 (29.2%)	<0.001
B2/B3	2 (7.1%)	17 (70.8%)	
Masaoka-Koga			
Stage I	9 (32.1%)	5 (20.8%)	0.53
Stage II/III	19 (67.9%)	19 (79.2%)	

*BMI* body mass index, *CT* computed tomography, *PD-L1* Programmed death ligand 1; *SUV* standard uptake value, *VATS* video-assisted thoracic surgery, *WHO* World health organization

SUVmax ≤3 was significantly linked to PD-L1<50% (p=0.046). Patients receiving adjuvant treatments also had higher PD-L1 levels (p<0.001). Furthermore, the relationship between histology and PD-L1 expression demonstrated a strong significance (p<0.001), with more aggressive histological types (B2, B3) associated with higher PD-L1 expression.

**Fig. 1:**
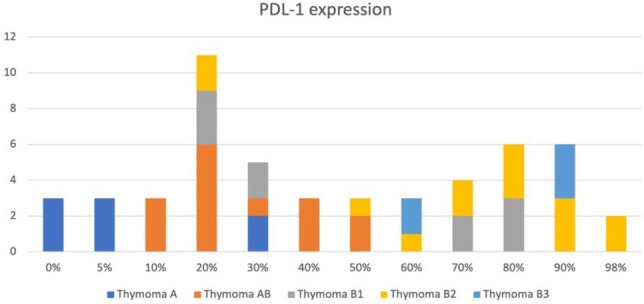
Programmed Death Ligand 1 (PD-L1) distribution across thymoma histotypes

**Fig. 2:**
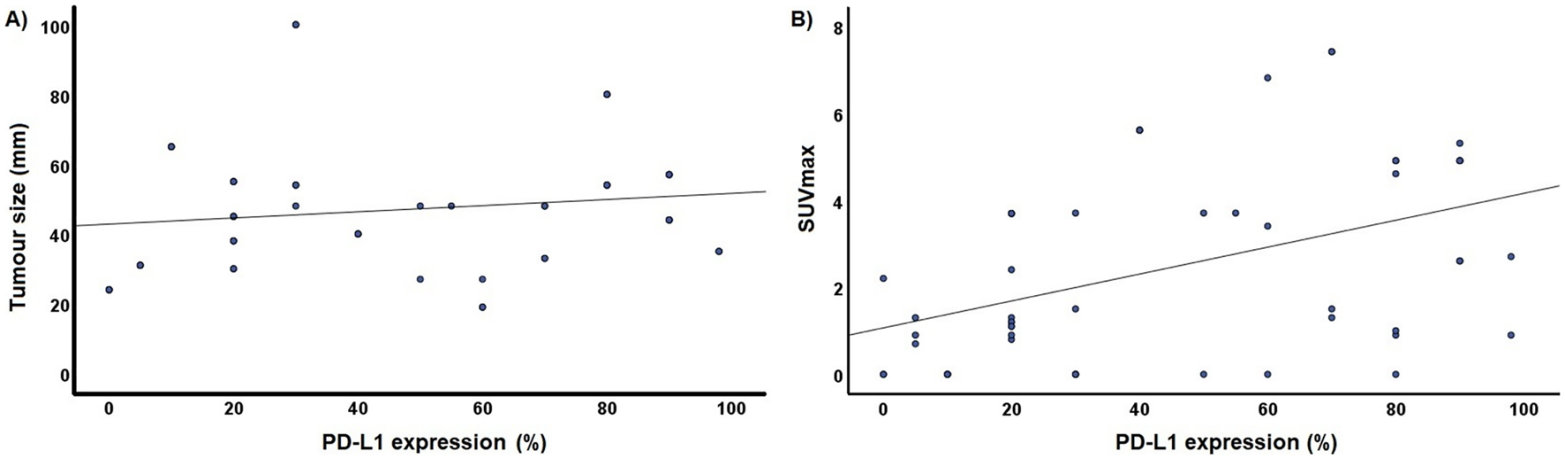
scatter plots showing correlation between Programmed Death Ligand 1 (PD-L1) and **A** tumour dimensions; **B** standard uptake value (SUV)max

Figure 1 illustrates the distribution of PD-L1 across thymoma histotypes. In thymoma type A, all patients (n=8) had PD-L1 <50%. For thymoma types AB, B1, and B2, PD-L1 ≥50% was observed in 13.3% (2/15), 50% (5/10), and 85.7% (12/14) of cases, respectively. In type B3, all patients (5/5) had PD-L1 ≥50%. These findings suggest that PD-L1 expression increases with histologic severity of thymoma, peaking in the aggressive B3 subtype. Figure 2 graphically represents the correlation between PD-L1 expression and tumour dimensions (Fig. 2A) and SUVmax (Fig. 2B).

### Survival analysis

During the follow-up period, a total of 12/52 patients (23.1%) experienced recurrence. All recurrences were local and involved the pleura. Among these patients, 3/12 (25.0%) have undergone thymectomy by VATS, 3/25 (25.0%) by sternotomy and 6/12 (50.0%) by thoracotomy. Thymomas with PD-L1 ≥50% presented a 5-year DFS rate of 54.2%, while patients with PD-L1 <50% had only one recurrence and a 5-year DFS rate of 96.4%. Cox regression analysis for DFS was reported in Table 3. Univariable analysis showed significant correlations with gender (p=0.010), comorbidities (p=0.050), smoking history (p=0.030), SUVmax (p=0.031), adjuvant treatment (p<0.001), WHO histology (p=0.017), Masaoka-Koga stage (p=0.018) and PD-L1 expression (p=0.007). No statistically significant correlation was found between recurrence and age, BMI, autoimmune diseases, tumour diameter or surgical approach.

**Table 3:** Cox regression analysis

	Disease free survival		
	Univariable	Multivariable	
	p-value	HR (95% CI)	p-value
Age (≤68 vs >68 years)	0.86	–	–
Gender (F vs M)	**0.010**	8.9 (0.8–105.2)	0.080
BMI (≤25 vs >25 kg/m2)	0.10	–	–
Comorbidity (no vs yes)	**0.050**	2.7 (0.5–13.9)	0.22
Autoimmune disease (no vs yes)	0.78	–	–
Myasthenia Gravis (no vs yes)	0.55	–	–
Treatment (surgery alone vs adjuvant)	**0.001**	0.1 (0.0–3.2)	0.19
Smoke history (no vs yes)	**0.030**	15.1 (0.3–24.6)	0.96
CT diameter (≤40 vs >40 mm)	0.57	–	–
SUVmax (≤3 vs >3)	**0.031**	3.2 (0.6–17.8)	0.19
Surgical approach (VATS vs open)	0.44	–	–
WHO Histology (A-AB-B1 vs B2-B3)	**0.017**	1.6 (1.1–8.9)	**0.044**
Masaoka-Koga stage (I vs II-III)	**0.018**	12.7 (2.6–19.0)	**0.026**
PDL-1 (<50% vs ≥50%)	**0.007**	5.4 (1.5–16.9)	**0.028**

Significant p-values are reported in bold

*BMI* body mass index, *CI* confidence interval, *CT* computed tomography, *F* female, *HR* hazard ratio, *M* male, *PD-L1* Programmed death ligand 1, *SUV* standard uptake value, *VATS* video-assisted thoracic surgery, *WHO* World health organization

At multivariable analysis, a higher expression of PD-L1 confirmed to be significantly correlated with worse DFS with an hazard ratio (HR) of 5.4 (95% confidence interval, CI, 1.5–16.9, p=0.028), only together with a higher grade at WHO histology classification (HR 1.6, 95% CI 1.1–8.9, p=0.044) and a higher Masaoka-Koga stage (HR 12.7, 95% CI 2.6–19.0, p=0.026).

**Fig. 3:**
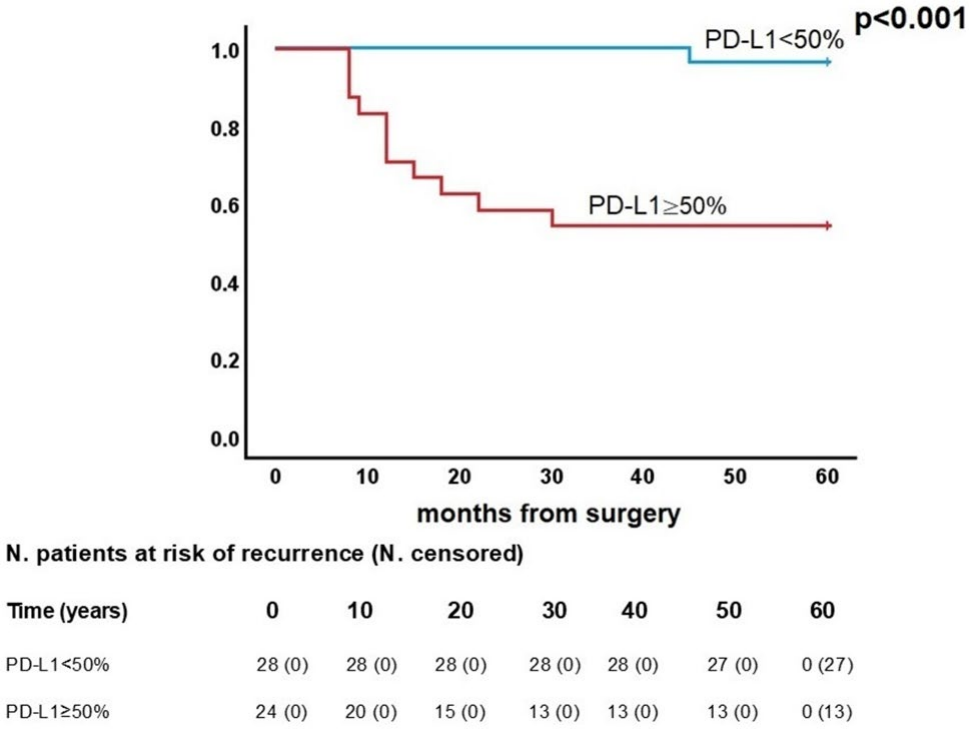
Kaplan-Meier curve for disease-free survival according to Programmed Death Ligand 1 (PD-L1)

**Fig. 4:**
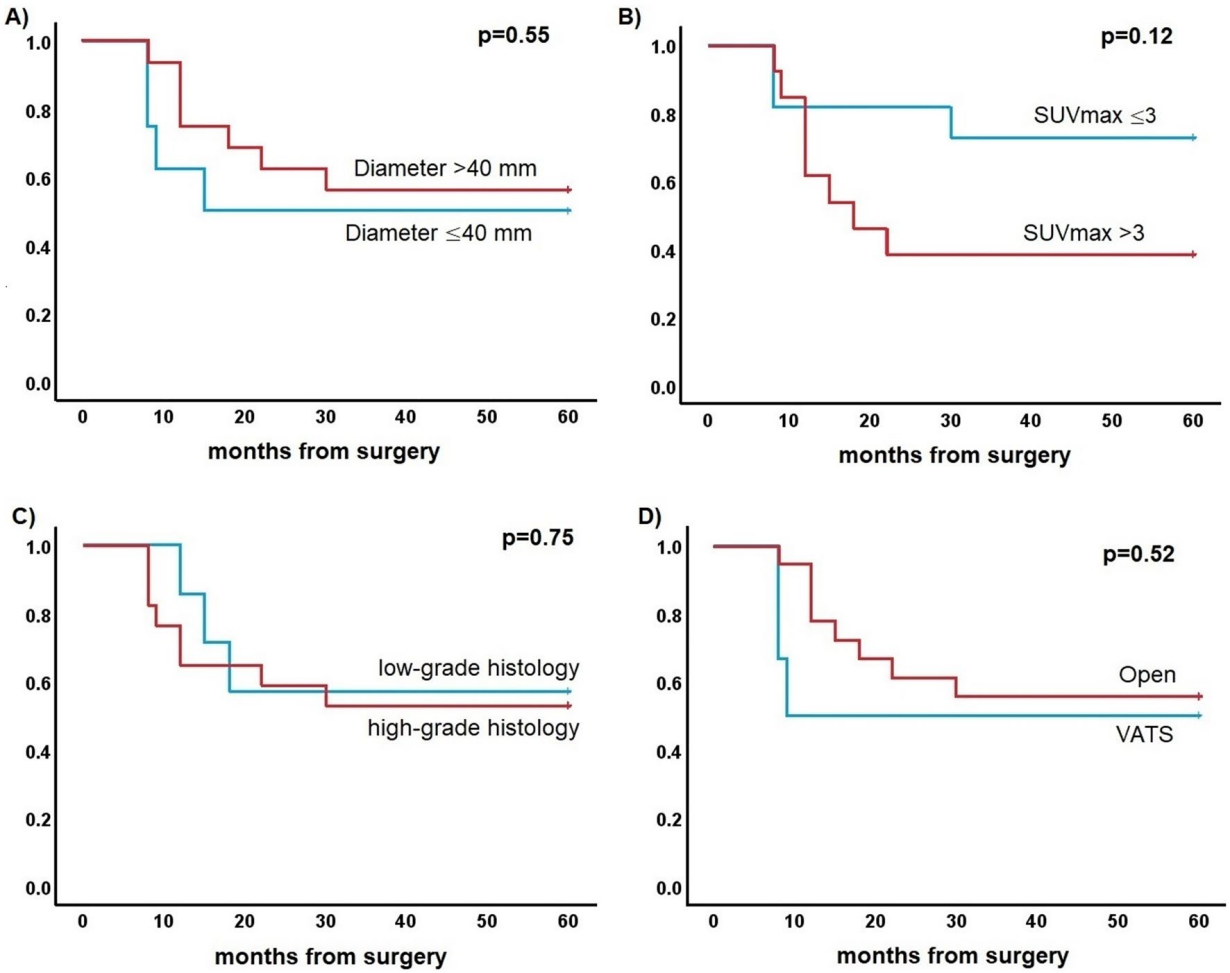
Kaplan-Meiers curve for disease-free survival among patients with Programmed Death Ligand 1 (PD-L1) ≥50% according to **A** tumour dimensions; **B** standard uptake value (SUV)max; **C** World Health Organization (WHO) histology; **D** surgical approach

Kaplan-Meier analysis (Figure 3) confirmed that higher PD-L1 expression was associated with significantly worse DFS (p<0.001). Subgroup Kaplan-Meier analysis among patients with PD-L1 ≥50% did not show a correlation between tumour dimensions or SUVmax, WHO histology or surgical approach and DFS (Fig. 4).

## Discussion

Over the past decade, technological advances have enabled the identification of a wide spectrum of molecular aberrations and altered signaling pathways in thymomas [20]. These insights have led to distinct molecular profiles and opened the door to new targeted therapies, with several phase 1 and 2 trials underway [21, 22].

Sakane et al. analysed the KRAS, HRAS, NRAS, EGFR, PIK3 CA, AKT1, BRAF, and TP53 genes in thymic carcinomas and thymomas [23]. They found mutations primarily in KRAS, TP53, HRAS, and EGFR in thymic carcinomas, while thymomas had mutations in PIK3 CA (6.1%), HRAS (3.03%), and AKT1 (3.03%) genes [23].

The role of immune checkpoint inhibitors has gained increasing attention as a potential therapeutic strategy for various malignancies [9, 24]. One of the most extensively studied biomarkers in thymic tumours is PD-L1, given its role in immune checkpoint pathways. However, its prognostic significance remains controversial [24].

In literature, even if some studies have reported high PD-L1 expression in thymomas (ranging from 68 to 94%) and thymic carcinomas (34–88%), conflicting findings exist regarding its association with clinical outcomes [7, 25, 26]. In 2015, Padda et al. analyzed 69 thymic epithelial tumours and they found significantly higher PD-L1 expression in thymomas compared to normal thymus tissue (68.1% vs. 17.6%, p=0.0036) [25]. The authors also highlighted as high PD-L1 thymomas correlated to a worse OS (HR 5.40, 95%CI 1.13–25.89, p=0.035) and DFS (HR 2.94, 95% CI 0.94–9.24, p=0.064) [25].

Conversely, Katsuya et al. examined a larger cohort of 141 thymic epithelial tumours, reporting high PD-L1 expression in 70% of thymic carcinoma samples but only in 23% of thymomas [27]. In addition, with reference to survival, they observed no significant change in OS among thymoma patients basing on PD-L1 expression [27].

Higher PD-L1 expression levels have been also associated with more aggressive histological subtypes, advanced Masaoka stages, and poorer clinical outcomes [11, 13, 25, 28, 29]. In particular, Yokoyama et al. specifically observed elevated PD-L1 levels in high-grade thymomas (B2/B3) and advanced stages III/IV (p=0.043) [30]. Interestingly, in their study PD-L1 expression increased after chemotherapy compared with levels before systemic treatment [31]. They also documented a correlation with worse DFS after surgery (p=0.021), even though not with OS (p=0.957) [31].

In 2020, a comprehensive meta-analysis by Koh et al. further supported the link between PD-L1 expression and poorer outcomes in thymic tumors [24]. Their study highlighted PD-L1 expression was linked to advanced stage of disease (OR 3.93, 95% CI 2.44–6.32, P < 0.001) and worse OS in thymoma (HR 1.89, 95% CI 1.09–3.28, P = 0.023) but not to DFS (HR 1.36, 95% CI: 0.97–1.92, P = 0.074) [24].

Consistent with these findings, our study revealed elevated PD-L1 expression in 85.7% of B2 thymomas and all B3 thymomas, as well as in those cases requiring adjuvant treatment. Notably, nearly all patients who experienced recurrence had PD-L1 expression exceeding 50%. Furthermore, multivariable Cox regression analysis identified high PD-L1 levels, along with histology and stage, as key indicators of poorer DFS prognosis. These results suggest an active role of immune evasion mechanisms in aggressive subtypes, aligning with observed poorer prognoses.

The correlation between higher PD-L1 expression and poor prognosis opens promising prospects for immunotherapy[32].

Cho et al. conducted a phase II study on pembrolizumab, showing partial responses in 28.6% of thymomas and 19.2% of thymic carcinomas, with stable disease observed in 71.6% of thymomas [32]. Rajan et al. also reported an objective response rate of 28.6% (95% CI, 8.2–64.1%) and 29% (95% CI, 3.7–71.0%) in trials testing pembrolizumab and avelumab in 14 patients with advanced thymoma [8, 21]. Additionally, Giaccone et al. found pembrolizumab achieving a 22.5% response rate, with a DFS of 4.2 months and an OS of 24.9 months, in recurrent thymic carcinoma, thus suggesting an advantage over chemotherapy [7].

However, immune-related adverse events were significant, emphasizing the need for further research on toxicity management. Despite promising results, the off-label status of immunotherapy adds complexity to its use in thymomas, requiring careful monitoring for adverse events. Pembrolizumab is currently an off-label option for patients who have undergone at least one platinum-based chemotherapy regimen [33].

Our study presents the obvious limitation of a retrospective analysis, thus making selection bias possible. The use of some quite dated histological samples could have altered and reduced the PD-L1 expression. The bicentric nature of the study could have introduced bias due to the management of histological samples by different pathologists. Moreover, the relatively small cohort of patients and the lack of controlled conditions also constrain the possibility to generalize the results and the application of our findings in clinical practice. However, it is important to emphasize that thymoma is a quite rare condition and that the results are concordant with other studies in literature and highlighted the potential role of PD-L1 as a prognostic marker. They reinforce the need for continued research into precision medicine for thymomas. Collaborative efforts across centers or access to larger databases would enhance the reliability and impact of future studies.

## Conclusions

The mutational landscape of thymomas remains a subject of ongoing exploration. Our study demonstrated that high PD-L1 expression is predominantly associated with aggressive thymoma histotypes (B2-B3) and correlates with poorer disease-free survival.

These results highlighted the potential effectiveness for targeted therapies in treating thymomas and reinforced the role of high PD-L1 expression as a prognostic biomarker. Indeed, according to our findings, patients with a high PD-L1 expression could be selected for adjuvant therapy despite the stage of disease or at least for closer follow-up. Further research is needed to clarify their molecular landscape and improve treatment approaches.

## Data Availability

All data generated or analysed during this study are included in this published article.
